# The effect of tobacco control policy on smoking cessation in relation to gender, age and education in Lithuania, 1994–2010

**DOI:** 10.1186/s12889-015-1525-8

**Published:** 2015-02-25

**Authors:** Jurate Klumbiene, Edita Sakyte, Janina Petkeviciene, Ritva Prattala, Anton E Kunst

**Affiliations:** Public Health Faculty, Lithuanian University of Health Sciences, Siaures av. 57, LT-49264 Kaunas, Lithuania; National Institute for Health and Welfare, Helsinki, Finland; Department of Public Health, Academic Medical Centre, University of Amsterdam, Amsterdam, the Netherlands

**Keywords:** Tobacco control policy, Smoking cessation, Quit ratio, Gender, Age, Education

## Abstract

**Background:**

This study aimed to evaluate the association between tobacco control policies and trends in smoking cessation according to gender, age and educational level in Lithuania in 1994–2010.

**Methods:**

The data were obtained from nine cross-sectional postal surveys conducted biennially within the framework of Finbalt Health Monitor project during 1994–2010. Each survey was based on a nationally representative random sample drawn from the National population register. The sample consisted of 3000 citizens aged 20–64 in 1994–2008 surveys and 4000 in the 2010 survey. In total, 17161 individuals participated in all surveys. The development of tobacco control policy in Lithuania was assessed using the Tobacco Control Scale (TCS). The association of the TCS scores with short-term and long-term quitting according to gender, age and education was examined using logistic regression analysis with control for secular trends.

**Results:**

Over the last two decades, a large improvement in the development of tobacco control policy has been achieved in Lithuania. At the same time, this progress was associated with the increase in smoking cessation. A significant increase in both short-term and long-term quit ratios was found among people aged 20–44. An increase by 10 points on the TCS was associated with 17% increase in the odds of short-term quitting and with 15% increase in the odds of long-term quitting. The association between tobacco control policies and long-term quitting was stronger among younger than older people. No differential effect of tobacco control policies on smoking cessation was found in relation to gender and educational level.

**Conclusions:**

The improvement in Lithuanian tobacco control policies was associated with an increase in smoking cessation in long-term perspective. These policies have not only benefitted highly educated groups, but lower educated groups as well. Nonetheless, further development of comprehensive tobacco control policies is needed in order to decrease social inequalities in smoking cessation.

**Electronic supplementary material:**

The online version of this article (doi:10.1186/s12889-015-1525-8) contains supplementary material, which is available to authorized users.

## Background

Tobacco smoking is one of the main factors contributing to premature mortality and morbidity in Europe. Lithuania has extremely high mortality rates from smoking-related diseases, such as cardiovascular diseases, cancers, and respiratory diseases [1,2]. In 2010, the age-standardized mortality rate from cardiovascular diseases was 120.6 per 100 000 Lithuanian population aged 0–64 years, while the average rate in the European Union (EU) was 43.4 per 100 000 population of the same age [2]. The proportion of deaths attributable to tobacco was 25% for Lithuanian men and 3% for women aged 30 years and more [3]. Furthermore, the studies undertaken in Lithuania have shown growing social inequalities in mortality [4-6]. The higher prevalence of smoking among people from lower socioeconomic groups may be one of the causes of higher mortality rate in those groups compared to people with higher socioeconomic position [7,8]. Reducing inequalities in smoking is therefore a key public health priority and tobacco control policies should be equity-oriented.

In the Soviet era there was no tobacco legislation, no formulated tobacco control policy, and no effective health education. Since regaining of independence in 1990, various activities were initiated in Lithuania to create a legal basis for tobacco control. In 1996, Lithuanian Parliament adopted the Law on Tobacco Control, which introduced a smoking ban in all enclosed workplaces, domestic trains as well as locations used for education, health care, administration and cultural activities [9]. An amendment to the Law on Tobacco Control came into force in 2007, extending the ban on smoking to restaurants, cafe and bars. In 2000, a complete ban on tobacco advertising came into force. Lithuania ratified World Health Organization (WHO) Framework Convention on Tobacco Control (FCTC) in 2004 and committed itself to implementing national legislation that is consistent with the FCTC [10]. After joining of the EU in 2004, Lithuania was obliged to change tobacco excise tax policy in order to meet the EU requirements. The rise in tobacco excise rates was moderate in 2004–2007, however, in 2009 specific excise tax increased sharply from 22.9 Euro to 38.2 Euro and ad-valorem excise tax increased from 15% to 25%. As a result, the prices of cigarettes grew slowly in 2004–2008, but they considerably increased in 2009 [11,12].

Several previous studies demonstrated that many of tobacco control policies are effective in reducing of smoking prevalence [13-15]. Analyses of Lithuanian Health Behaviour Monitoring showed that the prevalence of daily smoking was increasing up to the year 2000, especially among women. Since 2002, a decreasing trend has been observed among men and the increase of smoking prevalence levelled off among women [16,17]. The decline in smoking prevalence was more pronounced in highly educated men than in low educated. Moreover, the increase in smoking prevalence was lower among highly educated women compared to low educated [17].

Change in smoking prevalence is the result of two processes – initiation and cessation of smoking. The impact of tobacco control measures on socio-economic inequalities in smoking cessation has not been examined extensively. Some studies have shown widening inequalities in smoking cessation, while others have found that inequalities in smoking cessation did not change [18-21]. Moreover, there are scarce data about the impact of tobacco control measures on smoking outcomes and smoking inequalities in former Soviet republics where tobacco epidemic is following a somewhat different pattern that it took in the West [22]. In Soviet times, smoking prevalence was extremely high among men and very low among women. During transition period, in most former Soviet republics smoking rates in men failed to decline in ways predicted by the Western smoking epidemic model, while the increase in smoking rates among women was delayed [22,23].

The aim of the study was to evaluate the association between tobacco control policies and trends in smoking cessation in Lithuania in 1994–2010. The specific research objectives are: 1) to assess time trends in short-term and long-term quit ratios by gender, age and educational level between 1994 and 2010; 2) to evaluate the association between the development of Lithuanian tobacco control policy and the changes in quitting in 1996–2010; 3) to assess whether the associations of tobacco control policy development with short-term and long-term quitting depend on gender, age and educational level.

## Methods

### The assessment of tobacco control policy development in Lithuania

The development of tobacco control policy in Lithuania was assessed using the Tobacco Control Scale (TCS) [24]. Information on five tobacco control policy areas (price of cigarettes and other tobacco products, smoke-free work and other public places, comprehensive bans on advertising and promotion, large direct health warning labels, and treatment to help dependent smokers stop) was collected for each year between 1995 and 2010. The intensity of policies in each of five areas was quantified separately and the scores of all areas were summed (maximum possible score was 85). The information on smoke-free policies, bans on tobacco advertising and promotion, health warning labels was derived from Lithuanian Law on Tobacco Control with all its amendments, and from Tobacco Control Database for the WHO European Region [9,25].

The calculation of the TCS scores for tobacco prices was based on the approach recommended by the original TCS [24]. However, some modifications were introduced as the calculations were based on the prices of the lowest class of cigarettes in Lithuania. This class of cigarettes is more available and mostly used by lower socioeconomic groups. The data on tobacco prices were obtained from the database of Statistics Lithuania [12]. The tobacco prices of each year were divided by the Lithuanian Gross Domestic Product, expressed in Purchasing Power Standards per capita [12]. Those numbers were divided by the reference price which was the price of cigarettes in the UK in 2007 (6.6 Euro) [26]. As a result, a maximum of 30 points could be allocated to the reference price. The TCS scores for tobacco prices were calculated by multiplying the estimated ratio by 30 points.

### Health behaviour monitoring survey

#### Study population

The data were obtained from nine cross-sectional postal surveys conducted within the framework of Finbalt Health Monitor project [27]. In Lithuania, the surveys have been carried out every second year since 1994. Each survey was based on a nationally representative random sample drawn from the national population register. The sample consisted of 3000 individuals aged 20–64 in 1994–2008 surveys and 4000 individuals in the 2010 survey. The sampling unit was the individual in all surveys. No measures were taken to substitute non-respondents. The methodology and questionnaires were standardized across the survey years. The questionnaire has remained essentially unchanged over the years (Additional file 1). Every time it was mailed between April and June with one reminder. Response rates varied from 54 to 74% being lower in the last surveys. In total, 17161 individuals participated in all surveys. The Lithuanian Bioethics Committee approved all surveys. Written informed consent for participation was obtained from the respondents in all surveys.

#### Measurements

The smoking status of the respondents was obtained using the following questions: ‘Have you ever smoked?’, ‘Have you ever smoked at least 100 cigarettes?’, ‘Have you ever smoked daily at least one year?’ and ‘When did you last smoke?’ The respondents were classified as “daily smokers”; “occasional smokers”, “ex-smokers given up smoking 1–12 months ago”, “ex-smokers given up smoking more than one year ago”, and “never-smokers”. The individuals were considered as daily smokers when they smoked daily for at least one year and smoked in the day of filling in the questionnaire or a day before it. Those who answered that they had smoked daily at least one year and had stopped smoking were defined as ex-smokers. The respondents were considered as occasional smokers if they had indicated occasional smoking by themselves and had not smoked in the day of filling in the questionnaire or a day before it.

Two measures of smoking cessation were calculated: short-term and long-term quit ratio*.* The short-term quit ratio was calculated as the ratio of the number of ex-smokers who had given up smoking 1–12 months ago divided by the number of daily smokers plus ex- smokers who had given up smoking 1–12 months ago. The long-term quit ratio was calculated as the ratio of the number of ex-smokers given up smoking more than 12 months ago divided by the number of ever-smokers (daily smokers plus ex-smokers). Occasional smokers were excluded from the analysis. The proportion of occasional smokers was very stable in all surveys (7 to 9% in men and 6 to 8% in women).

The socio-demographic variables used in the analysis were: gender, age, and education. Age was categorized into two groups: 20–44 and 45–64. The respondents were also categorized into two groups according to their educational level: persons with low education (primary education, incomplete secondary education or secondary school), and persons with high education (college, vocational school or university).

#### Statistical analysis

Age-standardized quit ratios were calculated for each study year, gender and educational group. The data were age standardized using the European population of 2008 as standard. Normal approximation was used in the calculation of 95% confidence intervals for standardized rates.

The association between the TCS scores and quitting was examined using logistic regression analysis. The analysis was performed using pooled data from all surveys carried out in 1996–2010*.* The TCS scores of one year before the respective survey were included into logistic regression analysis. The TCS scores used in analysis were divided by 10 and treated as continuous variable. Dependent variables were short-term and long-term quitting. We fitted four models for each dependent variable. Model 1 included following variables: the TCS scores, gender, age and education. Men, the respondents aged 20–44 and those with low education were defined as reference groups. Model 2 additionally included terms for the interaction between the TCS scores and gender, to examine whether there were gender related differences in the association between the TCS scores and smoking quitting. The interaction between the TCS scores and age was added in the model 3 to assess age differences in the abovementioned association. Model 4 included the variables of model 1 and the interaction between the TCS scores and education level to assess educational differences in the association between the TCS scores and smoking quitting. All models were controlled for daily smoking prevalence at the time of the survey. Data were analysed using the statistical package SPSS (version 20).

## Results

**Figure 1:**
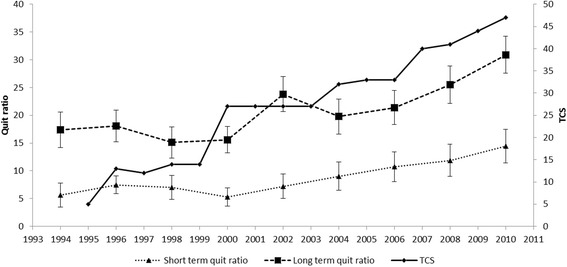
**The Tobacco Control Scale (TCS) scores and quit ratios in Lithuania from 1994 to 2010.** Short-term quit ratio = the number of ex-smokers given up smoking 1–12 months ago /daily smokers plus ex-smokers given up smoking 1–12 months ago. Long-term quit ratio = the number of ex-smokers given up smoking more than 12 months ago/ever smokers.

Table 1 shows annual TCS scores for each of the five tobacco control policies in Lithuania. The first increase in the TCS scores (by 8 points) was observed between 1995 and 1996 when the Law on Tobacco Control came into force (Table 1, Figure 1). In 1996–1998, the scores did not change. Between 1999 and 2000, the TCS scores increased significantly (by 13 points). This progress was related to the enforcement of a complete ban on tobacco advertising. In the period 2000 – 2006, the scores increased gradually reaching 33 in the year 2006. Between 2006 and 2007, the scores increased by 7 points due to ban of smoking in cafes and restaurants. The decrease in affordability of cigarettes resulted in the increase of the scores by 7 points from 2007 to 2010.

**Table 1:** **Annual tobacco control scores for tobacco control policies in Lithuania, 1995-2010**

**Year**	**Price of cigarettes and other tobacco products**	**Smoke free work and other public places**	**Comprehensive bans on advertising and promotion**	**Large direct health warning labels**	**Treatment to help dependent smokers stop**	**Total TCS score**
1995	4	0	0	1	0	5
1996	5	7	0	1	0	13
1997	4	7	0	1	0	12
1998	5	7	0	2	0	14
1999	5	7	0	2	0	14
2000	6	7	11	2	1	27
2001	6	7	11	2	1	27
2002	6	7	11	2	1	27
2003	6	7	11	2	1	27
2004	7	7	11	6	1	32
2005	7	7	11	6	2	33
2006	6	7	11	6	2	33
2007	6	15	11	6	2	40
2008	7	15	11	6	2	41
2009	11	15	11	6	1	44
2010	14	15	11	6	1	47

Table 2 presents the number and the percentage of ever-smokers and the distribution of ever-smokers by gender, age and education in each year of health behaviour survey. In 1994–2000, short-term quit ratios were stable (Figure 1). Since 2000, they have been constantly increasing. In 2010, the proportion of short-term ex-smokers was significantly higher as compared with the years 1994 to 2000 (p < 0.05). Long-term quit ratios rose significantly between the years 2000 and 2002 (from 16% to 23.8%, p < 0.05). Since 2004, the proportion of long-term ex-smokers increased reaching 30.1% in the year 2010.

**Table 2:** **Socio-demographic characteristics of study population in 1994-2010**

**Study year**	**Total number of respondents**	**Ever-smokers**							
		**N**	**%**	**Gender**		**Age**		**Education**	
				**Men (%)**	**Women (%)**	**20-44 (%)**	**45-64 (%)**	**Low (%)**	**High (%)**
1994	1864	516	27.7	81.9	18.1	64.6	35.4	49.4	50.6
1996	2021	691	34.2	79.1	20.9	62.9	37.1	50.6	49.4
1998	1874	654	34.9	73.5	26.5	70.8	29.2	46.4	43.6
2000	2195	876	39.9	72.9	27.1	65.5	34.5	47.4	52.6
2002	1883	706	37.5	72.2	27.8	54.5	45.5	38.5	61.5
2004	1822	616	33.8	67.7	32.3	63.0	37.0	40.3	59.7
2006	1739	654	37.6	65.7	34.3	59.8	40.2	41.7	58.3
2008	1763	641	36.4	65.0	35.0	56.9	43.1	54.1	45.9
2010	2000	729	36.5	55.9	44.1	56.4	43.6	53.2	46.8

N – Number of ever smokers (daily smokers + ex-smokers).

There were no significant changes in short-term quit ratios among men or women between the individual study years and over the whole study period (Table 3). The long-term quit ratio increased by 1.7 times in men and by 2.1 times in women from 1994 to 2010. The greatest increase in long-term quit ratio was observed in men between the years 2000 and 2002 (from 16.6% to 24.1%, respectively). The proportion of long-term ex-smokers in women was significantly higher in the year 2010 than in the earlier surveys.

**Table 3:** **Age-standardized short-term and long-term quit ratios (%) in men and women in 1994-2010**

**Study year**	**Short-term quit ratio**				**Long-term quit ratio**			
	**Men**		**Women**		**Men**		**Women**	
	**%**	**95% CI**	**%**	**95% CI**	**%**	**95% CI**	**%**	**95% CI**
1994	3.9	1.9-6.0	12.8	5.5-20.2	17.0	13.4-20.5	14.3	7.2-21.5
1996	6.3	4.1-8.5	11.9	6.1-11.7	17.2	14.1-20.4	19.8	13.3-26.2
1998	4.0	2.0-5.9	15.4	9.5-21.3	15.9	12.6-19.1	13.8	8.6-19.1
2000	4.5	2.7-6.2	7.4	3.8-11.1	16.6	13.7-19.5	14.3	9.8-18.8
2002	5.4	3.1-7.7	11.8	6.5-17.0	24.1	20.4-27.8	22.8	16.8-28.9
2004	7.8	4.9-10.7	11.5	6.6-16.4	20.3	16.4-24.2	18.1	12.8-23.5
2006	7.8	5.0-10.7	16.2	10.7-21.6	20.3	16.5-24.2	22.3	16.8-27.7
2008	9.6	6.4-12.9	16.0	10.5-21.5	24.8	20.6-29.0	24.6	19-30.2
2010	9.5	6.1-12.9	20.4	15.2-25.7	29.7	25.2-31.2	30.5	25.5-35.5

Short-term quit ratio = the number of ex-smokers given up smoking 1–12 months ago/daily smokers plus ex-smokers given up smoking 1–12 months ago.

Long-term quit ratio = the number of ex-smokers given up smoking more than 12 months ago/ever smokers.

Abbreviations: *CI* confidence interval.

Table 4 presents quit ratios in two age groups in 1994–2010. Over the period of sixteen years, short-term and long-term quit ratios increased significantly among respondents aged 20–44, but did not change in the older age group. In the younger age group the largest increase in long-term quit ratio was found between the years 2008 and 2010 (16.5% and 28.3% respectively).

**Table 4:** **Short-term and long-term quit ratios by age in 1994-2010**

**Study year**	**Short – term quit ratio**				**Long – term quit ratio**			
	**20-44**		**45-64**		**20-44**		**45-64**	
	**%**	**95% CI**	**%**	**95% CI**	**%**	**95% CI**	**%**	**95% CI**
1994	6.4	3.7-9.2	3.3	0.1-6.5	8.3	5.3-11.2	34.1	27.2-40.9
1996	7.4	4.8-9.9	8.1	4.1-12.0	11.5	8.5-14.4	29.3	23.7-32.8
1998	7.4	4.9-10.0	6.0	2.1-9.8	12.7	9.7-15.8	20.9	15.1-26.8
2000	5.5	3.5-7.4	4.8	2.0-7.6	11.0	8.4-13.5	24.4	19.6-29.3
2002	7.7	4.8-10.7	6.5	3.2-9.8	16.1	12.4-19.8	33.0	27.8-38.2
2004	9.2	6.0-12.4	8.3	4.1-12.6	16.0	12.3-19.6	26.3	20.6-32.1
2006	13.0	9.3-16.6	5.5	2.1-8.8	15.3	11.8-18.9	30.4	24.8-36.0
2008	13.3	9.5-17.1	9.1	4.8-13.5	16.5	12.7-20.3	37.3	31.6-43.0
2010	16.2	12.0-20.4	10.5	6.3-14.7	28.3	24.0-32.7	34.2	28.9-39.4

Short-term quit ratio = the number of ex-smokers given up smoking 1–12 months ago/daily smokers plus ex-smokers given up smoking 1–12 months ago.

Long-term quit ratio = the number of ex-smokers given up smoking more than 12 months ago/ever smokers.

Abbreviations: *CI* confidence interval.

No significant changes in short-term quit ratios between the study years were observed among people from either low or high educational group (Table 5). From 1994 to 2010, the increase in long-term quit ratio was found in both educational groups. In the first survey long-term quit ratios were similar among low and high educated people. In 2010, a statistically significant difference in long-term ratios between educational groups was observed (25.2% and 36% respectively).

**Table 5:** **Age-standardized short-term and long-term quit ratios by educational level in 1994-2010**

**Study year**	**Short – term quit ratio**				**Long – term quit ratio**			
	**Low education**		**High education**		**Low education**		**High education**	
	**%**	**95% CI**	**%**	**95% CI**	**%**	**95% CI**	**%**	**95% CI**
1994	4.3	1.6-7.0	7.2	3.7-10.6	14.1	9.8-18.4	18.7	14.0-23.4
1996	6.6	3.7-9.4	8.5	5.2-11.8	15.6	11.7-19.3	20.1	15.9-24.3
1998	8.0	4.5-11.3	6.3	3.8-8.8	13.2	9.3-17.0	17.3	13.2-21.3
2000	4.4	2.3-6.5	6.3	3.8-8.8	11.4	8.3-14.5	20.3	16.6-24.0
2002	4.5	1.7-7.4	8.9	5.8-12.1	22.3	17.3-27.3	24.7	20.5-28.8
2004	10.5	6.3-14.6	7.8	4.7-11.0	14.5	10.1-18.9	23.0	18.6-27.4
2006	10.7	6.7-14.7	10.4	6.8-14.0	15.4	11.1-19.7	25.1	20.7-29.5
2008	9.0	5.6-12.4	15.5	10.5-20.5	20.3	16.0-24.4	30.2	25.0-35.5
2010	11.3	7.7-15.0	18.0	12.9-23.2	25.2	20.9-29.5	36.0	30.8-41.1

Short-term quit ratio = the number of ex-smokers given up smoking 1–12 months ago/daily smokers plus ex-smokers given up smoking 1–12 months ago.

Long-term quit ratio = the number of ex-smokers given up smoking more than 12 months ago/ever smokers.

Abbreviations: *CI* confidence interval.

The association between the TCS scores with quitting was estimated by logistic regression analysis (Tables 6 and 7). An increase by 10 points on the TCS scale was associated with an increase of 17% in the odds of short-term quitting (Model 1 in Table 6). Short-term quitting was more common among women than men and among younger than older individuals. No interactions between the TCS scores and gender, age or education were observed (Models 2–4).

**Table 6:** **The association of Tobacco Control Scale scores and socio-demographic variables with short-term quitting in 1996–2010**

**Independent variables**		**Model 1**	**Model 2**	**Model 3**	**Model 4**
TCS	OR	**1.17**	**1.19**	**1.22**	**1.13**
	95% CI	1.07-1.27	1.07-1.33	1.10-1.34	1.01-1.27
Gender (women vs men)	OR	**2.10**	**2.44**	**2.12**	**2.09**
	95% CI	1.70-2.59	1.56-3.83	1.71-2.62	1.70-2.58
Age (45–64 vs 20–44)	OR	**0.76**	**0.77**	1.16	**0.76**
	95% CI	0.61-0.96	0.61-0.96	0.71-1.90	0.60-0.95
Education (high vs low)	OR	1.20	1.20	1.22	1.01
	95% CI	0.97-1.47	0.97-1.47	0.99-1.50	0.65-1.57
Gender × TCS	OR		0.94		
	95% CI		0.81-1.10		
Age × TCS	OR			0.85	
	95% CI			0.72-1.01	
Education × TCS	OR				1.07
	95% CI				0.92-1.24

Model 1 included TCS scores, gender, age and education; model 2 – the variables of the model 1 and the interaction between TCS scores and gender; model 3 - the variables of the model 1 and the interaction between TCS scores and age; model 4 - the variables of the model 1 and the interaction between TCS scores and education; all models were adjusted for the prevalence of daily smoking.

OR which differ from 1.00 with statistical significance (p < 0.05) are presented in bold.

Abbreviations: *TCS* - Tobacco Control Scale**,** *OR* - odds ratio, *CI* - confidence interval.

**Table 7:** **The association of Tobacco Control Scale scores and socio-demographic variables with long-term quitting in 1996–2010**

**Independent variables**		**Model 1**	**Model 2**	**Model 3**	**Model 4**
TCS	OR	**1.15**	**1.16**	**1.3**	**1.16**
	95% CI	1.09-1.21	1.09-1.23	1.20-1.4	1.07-1.25
Gender (women vs men)	OR	1.05	1.06	1.05	1.05
	95% CI	0.91-1.21	0.91-1.22	0.91-1.21	0.91-1.21
Age (45–64 vs 20–44)	OR	**2.47**	**2.47**	**2.57**	**2.47**
	95% CI	2.17-2.81	2.17-2.81	2.26-2.93	2.17-2.82
Education (high vs low)	OR	**1.64**	**1.64**	**1.69**	**1.64**
	95% CI	1.44-1.87	1.44-1.87	1.48-1.93	1.44-1.87
Gender × TCS	OR		0.97		
	95% CI		0.87-1.09		
Age × TCS	OR			**0.79**	
	95% CI			0.71-0.87	
Education × TCS	OR				0.98
	95% CI				0.89-1.09

Model 1 included TCS scores, gender, age and education; model 2 – the variables of the model 1 and the interaction between TCS scores and gender; model 3 - the variables of the model 1 and the interaction between TCS scores and age; model 4 - the variables of the model 1 and the interaction between TCS scores and education; all models were adjusted for the prevalence of daily smoking.

OR which differ from 1.00 with statistical significance (p < 0.05) are presented in bold.

Abbreviations: *TCS* - Tobacco Control Scale, *OR* - odds ratio, *CI* - confidence interval.

The odds of long-term quitting increased by 15% with the 10 points increase of the TCS scores (Model 1 in Table 7). Likelihood of long-term quitting was higher in older than younger individuals and in highly educated compared to low educated. The logistic regression analysis did not show an interaction between the TCS scores and gender or educational level on long-term quitting (Models 2 and 4). Interaction between age and TCS scores on long-term quitting was significant (OR = 0.79; 95% CI: 0.71-0.87) (Model 3). Further analysis showed that in the younger age group, the increase by 10 points on TCS scale was associated with 30% increase in the odds of long-term quitting (OR = 1.30; 95% CI: 1.20-1.40) (data are not shown). The increase of odds in the older age group was smaller and not significant (OR = 1.03; 95% CI: 0.96-1.11).

## Discussion

Since 1995, a great progress in the development of tobacco control policy has been achieved in Lithuania. This progress was associated with an increase in smoking cessation between 1994 and 2010. A significant increase in both short-term and long-term quit ratios was found among people aged 20–44. The proportion of long-term quitters increased in men and women, and in both educational groups. The association between tobacco control policies and long-term quitting was statistically significant; this association was stronger among younger than older people. No differential effect of tobacco control policies on smoking cessation was found in relation to gender and educational level.

Our study had some important strengths. It used data on trends in smoking cessation among Lithuanian adult population over almost two decades. These data were derived from nine cross-sectional surveys conducted on nationally representative samples following the same methodology. The questions regarding smoking remained unchanged in all surveys ensuring comparability of data over a whole observational period. Information on the period of abstinence from smoking was available which allowed calculating short-term and long-term quit ratios. Short-term ratios could be more sensitive indicator for evaluation of tobacco control policy.

Some limitations of the study also need to be discussed. The data used in this study were based on self-report about smoking cessation without biochemical validation. An over-reporting of quitting could be possible, especially among short-term ex-smokers who gave up smoking 1–12 months ago. Some of short-term ex-smokers may have relapsed shortly after the survey. Since there can be differences in over-reporting of smoking cessation between the study years, age and educational groups, this could have influenced our results. Moreover, the number of ever-smokers was relatively small affecting the statistical power to detect the effect of interactions between the analysed variables, especially in women.

The next limitation could be a decreasing participation rate in the most recent surveys. In all surveys, women and older people responded better than men and younger individuals, but the decrease of response rate was almost similar in all gender and age groups. There is no information about educational level and smoking status of non-respondents. However, the distribution of study participants by educational level did not differ from that of Lithuanian population of the same age, suggesting that response rate was not related to educational level [28]. Other studies found that the prevalence of smoking was slightly higher among non-respondents [29,30]. To the best of our knowledge, there is no data about the differences in smoking cessation rates between participants and non-participants in postal surveys. The comparison of early and late respondents in Finbalt Health Monitor surveys discovered only slight differences in health behaviours, including smoking habits [31]. Bearing this in mind, we think that a decreasing response rate could not seriously bias the results of our study.

We found that quit ratios increased only among younger people (20–44 years old) and that the effect of tobacco control policies on quitting was stronger in this age group compared to older individuals. These findings are in line with some other studies showing higher impact of tobacco control measures on smoking cessation rates among young adults [19,32,33]. In the study period, Lithuania implemented mostly population-based measures: bans on tobacco advertising and smoking in the café, bars and restaurants as well as the increase in cigarettes prices. Smoking cessation services are extremely limited in this country, and there is no reimbursement of smoking cessation pharmacotherapy. The observed age differences in trends of smoking cessation could be due to the higher exposure of young people to the population-based measures mentioned above [19]. The data of Lithuanian health behaviour monitoring showed that young people, especially men, were more supportive to a ban on smoking in cafés, bars and restaurants [34]. For older people, the possible period of long-term quitting is much longer than for younger age group, which likely explains why no association was found between tobacco control policies in 1996–2010 and long-term quitting for older age group.

In our study, short-term quitting was more common among women than among men while there was no gender difference in long-term quitting. Furthermore, the impact of tobacco control measures on smoking cessation was similar both in men and women. In Soviet times, smoking prevalence in Lithuania was traditionally low among women, because it was socially unacceptable [35]. Since regaining of independence in the 1990, transnational tobacco industry started aggressive and sophisticated marketing of tobacco products targeting mainly women and young people. Women smoking became more socially accepted. These developments contributed to the increasing trends of smoking prevalence among women in 1994–2000 [17]. Strengthening of tobacco control activities since 2000 may have contributed to stabilization of smoking prevalence among women and to an increase of quitting.

We did not find a differential effect of tobacco control policies on smoking cessation in relation to educational level. These results are comparable with earlier findings from a study in 18 European countries which did not reveal consistent difference between higher and lower educated people regarding the association between smoking cessation and the TCS scores [18]. The implementation of smoke-free policy in Italy was immediately followed by the increase in quit ratios among both highly and low-educated men as well as among low-educated women [21]. Previous research suggests that various components of tobacco control policy might have a different impact on smoking cessation among low and high educated people. Tobacco tax and price increase may have larger effects among smokers with lower education, while partial smoke-free legislation and ban on tobacco advertising may have larger effects among smokers with high education [36-39].

In Lithuania, the implementation of tobacco control policies was followed by intensive discussions in mass-media. The findings from the longitudinal International Tobacco Control Europe Surveys suggested that anti-tobacco information campaigns might reduce the social acceptability of smoking [40]. Moreover, smoke-free legislation limiting the places where smoking is allowed might also decrease the social acceptability of smoking [41]. One of the indicators of the social acceptability of smoking is the proportion of smoke-free homes [42]. The data of Finbalt Health Monitor showed that over the last decade, the prevalence of passive smoking at home almost halved in Lithuania [43]. This decrease occurred among low educated as well as among high educated people indicating that smoking became less socially acceptable in the Lithuanian population, independent of educational level. Several studies have shown that the decreased social acceptability of smoking was associated with the increase in quitting intentions and smoking cessation [41,44]. Thus, the increase in long-term quit ratio among low and high educated individuals in Lithuania might be partially explained by the reduced social acceptability of smoking in society.

## Conclusions

Our results suggest that the progress in the Lithuanian tobacco control policies was associated with an increase of smoking cessation in long-term perspective. The effect of tobacco control policies was stronger among younger than older people, but it was similar among high and low educated smokers. This implies that tobacco control policies as developed in Lithuania in the 2000’s have not only benefitted highly educated groups, but lower educated groups as well. The Lithuanian experience does not support the fear that recent tobacco control policies may have widened inequalities in smoking outcomes. Nonetheless, further development of comprehensive tobacco control policies is needed in order to decrease social inequalities in smoking cessation.

## Additional file

Additional file 1:
**Smoking questionnaire.** The questionnaire used in the study is presented in Additional file 1.

## Abbreviations

EU: European Union
TCS: Tobacco control scale
WHO: World Health Organization
