# Supplementary material for: The effect of tobacco control policy on smoking cessation in relation to gender, age and education in Lithuania, 1994–2010
Source: BMC Public Health. 2015 Feb 25;15:181. doi: 10.1186/s12889-015-1525-8 (PMC4349467; doi:10.1186/s12889-015-1525-8)
Supplement: Additional file 1: — Smoking questionnaire. The questionnaire used in the study is presented in Additional file 1. [file 12889_2015_1525_MOESM1_ESM.pdf]

LITHUANIAN HEALTH BEHAVIOUR MONITOR

SMOKING QUESTIONNAIRE

1. **Sex**
   1 male
   2 female

2. **Year of birth** 19 ⌴⌴

3. **Education**

   1 Primary school
   2 Incompleted secondary school
   3 Secondary school
   4 Vocational school
   5 University

4. **How many hours a day do you spend at your workplace where somebody smokes?**
   1 more than 5 hours
   2 between 1-5 hours
   3 less than one hour a day
   4 almost never
   5 I do not work outside the home

5. **Do you or any family members smoke at home?**
   1 nobody smokes
   2 yes, somebody smokes

6. **Have you ever smoked in your life?**
   1 no
   2 yes

7. **Have you ever smoked at least 100 cigarettes, cigars or pipefuls in your lifetime?**
   1 no (proceed to question 9)
   2 yes

8. **Have you ever smoked daily (=almost every day for at least one year)? If so, how many years altogether?**
   1 no
   2 yes, I have smoked daily for a total of ⌴⌴ years

9. **Do you smoke at the present time (cigarettes, cigars, pipe)?**
   1 yes, daily
   2 occasionally
   3 not at all

10. **When did you last smoke?** If you smoke currently, please circle alternative 1.
    1 yesterday or today
    2 2 days - 1 month ago
    3 1 month - half a year ago
    4 half a year to one year ago

5 1-5 years ago
6 5-10 years ago
7 more than ten years ago

11. How much do you smoke or did you smoke before you stopped, on an average per day?

Please give an answer to each item.

filter cigarettes	└─┴─┘	cigarettes per day
non-filter cigarettes	└─┴─┘	cigarettes per day
self-rolled cigarettes	└─┴─┘	cigarettes per day
pipe	└─┴─┘	pipefuls a day
cigars	└─┴─┘	cigars a day
